# Author Correction: Microglia regulate central nervous system myelin growth and integrity

**DOI:** 10.1038/s41586-024-07696-3

**Published:** 2024-07-03

**Authors:** Niamh B. McNamara, David A. D. Munro, Nadine Bestard-Cuche, Akiko Uyeda, Jeroen F. J. Bogie, Alana Hoffmann, Rebecca K. Holloway, Irene Molina-Gonzalez, Katharine E. Askew, Stephen Mitchell, William Mungall, Michael Dodds, Carsten Dittmayer, Jonathan Moss, Jamie Rose, Stefan Szymkowiak, Lukas Amann, Barry W. McColl, Marco Prinz, Tara L. Spires-Jones, Werner Stenzel, Karen Horsburgh, Jerome J. A. Hendriks, Clare Pridans, Rieko Muramatsu, Anna Williams, Josef Priller, Veronique E. Miron

**Affiliations:** 1 UK Dementia Research Institute at The University of Edinburgh, Edinburgh, UK; 2 Centre for Discovery Brain Sciences, Chancellor’s Building, The University of Edinburgh, Edinburgh, UK; 3 Medical Research Council Centre for Reproductive Health, The Queen’s Medical Research Institute, The University of Edinburgh, Edinburgh, UK; 4 Centre for Clinical Brain Sciences, Chancellor’s Building, The University of Edinburgh, Edinburgh, UK; 5 Centre for Regenerative Medicine, Institute for Regeneration and Repair, The University of Edinburgh, Edinburgh, UK; 6 Departments of Molecular Pharmacology, National Institute of Neuroscience, National Center of Neurology and Psychiatry, Kodaira, Japan; 7 Department of Immunology and Infection, Biomedical Research Institute, Hasselt University, Hasselt, Belgium; 8 University MS Centre, Hasselt University, Hasselt, Belgium; 9 Barlo Multiple Sclerosis Centre, St Michael’s Hospital, Toronto, Ontario Canada; 10 Keenan Research Centre for Biomedical Science, St Michael’s Hospital, Toronto, Ontario Canada; 11 Department of Immunology, The University of Toronto, Toronto, Ontario Canada; 12 Wellcome Trust Centre for Cell Biology, King’s Buildings, The University of Edinburgh, Edinburgh, UK; 13 Biological and Veterinary Services, Chancellor’s Building, The University of Edinburgh, Edinburgh, UK; 14 Department of Neuropathology and Neurocure Clinical Research Center, Charité-Universitätsmedizin Berlin, Berlin, Germany; 15 Institute of Neuropathology, Centre for Basics in NeuroModulation, Faculty of Medicine, University of Freiburg, Freiburg, Germany; 16 Signalling Research Centres BIOSS and CIBSS, University of Freiburg, Freiburg, Germany; 17 Centre for Inflammation Research, The Queen’s Medical Research Institute, The University of Edinburgh, Edinburgh, UK; 18 Simons Initiative for the Developing Brain, Centre for Discovery Brain Sciences, University of Edinburgh, Edinburgh, UK; 19 Muir Maxwell Epilepsy Centre, University of Edinburgh, Edinburgh, UK; 20 Department of Psychiatry and Psychotherapy, Klinikum rechts der Isar, School of Medicine, Technical University of Munich, Munich, Germany; 21 Neuropsychiatry and Laboratory of Molecular Psychiatry, Charité-Universitätsmedizin Berlin and DZNE, Berlin, Germany

**Keywords:** Microglia, Oligodendrocyte

Correction to: *Nature* 10.1038/s41586-022-05534-y Published online 14 December 2022

In the version of this article originally published, Figs. 1b, 2f, and 2n contained errors. Figure 1b had accidental merging of the same Hoechst image with the Tmem119 stain for both genotypes. Figure 2f had incorrect statistical results which had not been updated following quantification of a larger number of axons. Figure 2n, a magnified inset of Fig. 2l in *Fire*^∆/∆^, had been rotated 180° from the lower magnification image in 2l and the green asterisks were too small. These figures have now been corrected, and the original and revised versions can be seen below in Figs. 1 and 2.

**Fig. 1:**
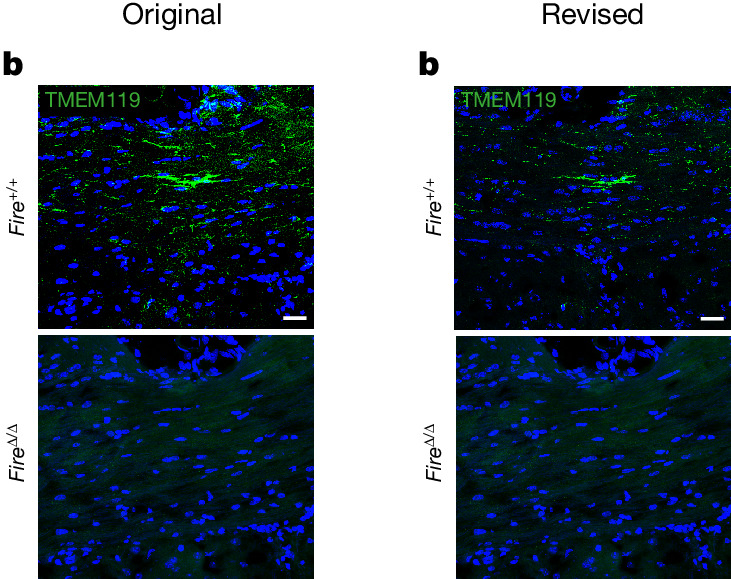
Original and revised Fig. 1b .

**Fig. 2:**
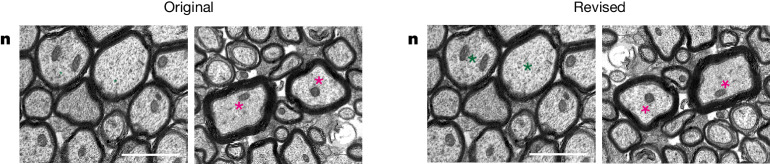
Original and revised Fig. 2n .

In addition, Extended Data Fig. 7f had a typographical error in the legend, which should have read *n* = 3 mice on normal diet and *n* = 4 mice on PLX diet. The source data for Fig. 2j provided were for ‘total myelinated axons’ but should have been for ‘normally myelinated axons’, and the correct data were provided in the tab for Fig. 2k.

We have now included the background strain of the *Fire*^∆/∆^ mice in the methods section.

Please note that the same representative images for IBA1 staining are included in both Fig. 4a and Extended Data Fig. 8a, yet this is not an error as these address different questions: Fig. 4a indicates total IBA1+ cells and Extended Data Fig. 8a indicates the subset of these which are LYVE1+ (perivascular macrophages).

