# Supplementary material for: Microglia regulate central nervous system myelin growth and integrity
Source: Nature. 2022 Dec 14;613(7942):120–9. doi: 10.1038/s41586-022-05534-y (PMC9812791; doi:10.1038/s41586-022-05534-y)
Supplement: Supplementary file 1 — Reporting Summary [file 41586_2022_5534_MOESM1_ESM.pdf]

nature portfolio

Corresponding author(s): MIRON

Last updated by author(s): Oct 17, 2022

Reporting Summary

Nature Portfolio wishes to improve the reproducibility of the work that we publish. This form provides structure for consistency and transparency in reporting. For further information on Nature Portfolio policies, see our Editorial Policies and the Editorial Policy Checklist.

Statistics

For all statistical analyses, confirm that the following items are present in the figure legend, table legend, main text, or Methods section.

n/a	Confirmed	
☐	☒	The exact sample size (*n*) for each experimental group/condition, given as a discrete number and unit of measurement
☐	☒	A statement on whether measurements were taken from distinct samples or whether the same sample was measured repeatedly
☐	☒	The statistical test(s) used AND whether they are one- or two-sided<br>*Only common tests should be described solely by name; describe more complex techniques in the Methods section.*
☒	☐	A description of all covariates tested
☐	☒	A description of any assumptions or corrections, such as tests of normality and adjustment for multiple comparisons
☐	☒	A full description of the statistical parameters including central tendency (e.g. means) or other basic estimates (e.g. regression coefficient) AND variation (e.g. standard deviation) or associated estimates of uncertainty (e.g. confidence intervals)
☐	☒	For null hypothesis testing, the test statistic (e.g. *F*, *t*, *r*) with confidence intervals, effect sizes, degrees of freedom and *P* value noted<br>*Give P values as exact values whenever suitable.*
☒	☐	For Bayesian analysis, information on the choice of priors and Markov chain Monte Carlo settings
☒	☐	For hierarchical and complex designs, identification of the appropriate level for tests and full reporting of outcomes
☒	☐	Estimates of effect sizes (e.g. Cohen's *d*, Pearson's *r*), indicating how they were calculated

Our web collection on statistics for biologists contains articles on many of the points above.

Software and code

Policy information about availability of computer code

Data collection	OpenEpi.com was used for power calculations for sample size. Leica SPE or Zeiss LSM 510 microscopes/software were used for confocal imaging. Human tissue was acquired using the Zeiss Zen2 (blue edition) software on a Zeiss AxioScan Z.1 SlideScanner. Electron micrographs were acquired on a Zeiss 9P02 electron microscope or Zeiss Gemini 300 scanning electron microscope. Single cells were processed on the 10X Chromium single cell platform and RNA sequences were read on a NovaSeq6000. Lipidomics were performed using the Nexera X2 UHPLC system and hybrid triple quadruple/linear ion trap mass spectrometer (6500+ QTRAP system; AB SCIEX). Flow cytometry was performed using a BD LSR Fortessa. Behavioural data was acquired using a Barnes Maze (San Diego instruments).
Data analysis	Cell counts were performed using Fiji/Image J software (Fiji.sc) and colocalization performed using Imaris software v9.7. Data handling was performed using Microsoft Excel 2016 and GraphPad Prism v.8 and v.9 were used for statistical analyses. Human electron micrograph images were stitched together using Fiji and QuPath 0.3.0 softwares. RNA sequencing analysis involved use of 10X Genomics Cell Ranger v5.0.0 pipeline to align the reads to the reference genome, using the mm10 genome supplied by 10X Genomics (refdata-gex-mm10-2020-A), R v4.1.1 to run the code, , SingleCellExperiment v1.14.1 to handle single cell experiment objects in R, scater v1.20.1 for quality control and producing plots, scran v1.20.1 for normalisation, batchelor v1.8.0 for the batch correction with fastMNN, clustree v0.4.4 to visualise the clustering, Seurat v4.1.0 for differential gene expression, ShinyCell v2.1.0 to produce an interactive app, org.Mm.eg.db v3.13.0 to annotate genes, ggplot2 v3.3.5 to perform custom plots, here v1.0.1 to ensure reproducible paths, and Matrix v1.3.4 for handling sparse matrices. Flow cytometry data was analysed on FCS express v.7. Behavioural data was assessed using Anymaze for video tracking (Stoelting Europe v4.9.9).

For manuscripts utilizing custom algorithms or software that are central to the research but not yet described in published literature, software must be made available to editors and reviewers. We strongly encourage code deposition in a community repository (e.g. GitHub). See the Nature Portfolio guidelines for submitting code & software for further information.

Data

Policy information about availability of data

All manuscripts must include a data availability statement. This statement should provide the following information, where applicable:

- Accession codes, unique identifiers, or web links for publicly available datasets
- A description of any restrictions on data availability
- For clinical datasets or third party data, please ensure that the statement adheres to our policy

The data that support the findings from this study are available from the corresponding author upon request as source data associated with this manuscript. Raw single cell RNA sequences datasets have been deposited in the Gene Expression Omnibus, accession code GSE215440. Sequencing Analyzed oligodendrocyte sequencing data is perusable on the following shiny app: https://annawilliams.shinyapps.io/shinyApp_oligos_VM/. Full details to replicate the analysis pipelines can be found in code scripts available on GitHub (https://github.com/Anna-Williams/Veronique-Firemice). Alignment to the reference genome, feature counting and cell calling was performed following the 10X Genomics CellRanger (v5.0.0) pipeline, using the default mm10 genome supplied by 10X Genomics (https://cf.10xgenomics.com/supp/cell-exp/refdata-gex-mm10-2020-A.tar.gz).

Human research participants

Policy information about studies involving human research participants and Sex and Gender in Research.

Reporting on sex and gender	This study used post-mortem tissue of human brain from non-neurological controls and people with ALSP. For controls, this consisted 4 males and 1 female. The ALSP cases consisted of 2 males and 3 females. All information pertaining to these tissues is listed in Extended Data Table 1.
Population characteristics	5 non-neurological controls died of rectosigmoid adenocarcinoma, metastatic gastric carcinoma, ischaemic heart disease/coronary artery atherosclerosis, and myocardial infarction/coronary artery atherosclerosis, and ranged from 34 to 58 years old. ALSP cases consisted of 3 who died of ALSP, 1 who died of pulmonary embolism, and 1 of bacterial pneumonia, and ranged from 22 to 58 years old. All information pertaining to these tissues is listed in Extended Data Table 1.
Recruitment	The tissue was obtained with full ethical approval from the Queen Square Brain Bank for Neurological Disorders, UCL Queen Square Institute of Neurology, the Department of Neuropathology at Charité-Universitätsmedizin Berlin and the Medical Research Council Edinburgh Brain and Tissue Bank (EBTB), and their use was in accord with the terms of the informed consents.
Ethics oversight	Ethical approval was granted to the Queen Square Brain Bank for the use of tissue by the National Health Service Health Research Authority through the London Central Research Ethics Committee.

Note that full information on the approval of the study protocol must also be provided in the manuscript.

Field-specific reporting

Please select the one below that is the best fit for your research. If you are not sure, read the appropriate sections before making your selection.

☒ Life sciences ☐ Behavioural & social sciences ☐ Ecological, evolutionary & environmental sciences

For a reference copy of the document with all sections, see nature.com/documents/nr-reporting-summary-flat.pdf

Life sciences study design

All studies must disclose on these points even when the disclosure is negative.

Sample size	For in vivo experiments, n=3-7 mice per genotype per time point. For human tissue, n=2-3 cases per condition. For mouse studies, sample size was determined using power calculations using OpenEpi.com, to reach a power of >80% at 0.05 significance. For human studies, no sample size calculation was performed as ALSP is an extremely rare disorder and we obtained as many cases as were available for our purposes.
Data exclusions	In the Barnes Maze data, 1 wildtype and 1 FIRE knockout mouse were excluded due to refusal to enter the escape hole during the training phase. This is indicated in the methods section.
Replication	All attempts at replication were successful
Randomization	Mice were randomly assigned to time points of assessment. For human studies, randomization was not involved nor applicable, as all tissue samples of a group were submitted to the same analyses (e.g. all resin blocks were submitted to EM processing, all frozen tissue to immunostaining).
Blinding	All manual counts were performed in a blinded manner. All behaviour experiments were performed in a blinded manner from data collection through to completion of data analysis. All analyses on human tissue were performed in a blinded manner.

Reporting for specific materials, systems and methods

We require information from authors about some types of materials, experimental systems and methods used in many studies. Here, indicate whether each material, system or method listed is relevant to your study. If you are not sure if a list item applies to your research, read the appropriate section before selecting a response.

Materials & experimental systems

n/a	Involved in the study
☐	☒ Antibodies
☒	☐ Eukaryotic cell lines
☒	☐ Palaeontology and archaeology
☐	☒ Animals and other organisms
☒	☐ Clinical data
☒	☐ Dual use research of concern

Methods

n/a	Involved in the study
☒	☐ ChIP-seq
☐	☒ Flow cytometry
☒	☐ MRI-based neuroimaging

Antibodies

Antibodies used	The following antibodies were used: α-MBP (AbD Serotec, 1:250; MCA409S, clone 12), α-MAG (Millipore, 1:100; MAB1567, clone 513), α-MOG (Millipore, 1:100; MAB5680, clone 8-18C5), α-CNPase (Sigma-Aldrich, 1:100; AMAB91072, clone CL2887), α-TMEM119 (Abcam, 1:100; ab209064, clone 28-3), α-IBA1 (Abcam, 1:500; ab5076), α-CD206 (Abcam, 1:100; ab64693), α-CD31 (R&D Systems, 1:100; AF3628), α-LYVE1 (Abcam, 1:100; ab14917), α-OLIG2 (Millipore, 1:100; AB9610), α-OLIG2 (Millipore, 1:100; MABN50, clone 211F1.1), α-APC ('CC1'; Abcam, 1:100; ab16794, clone CC1), α-SOX9 (Millipore, 1:500; AB5535), α-GFAP (Cambridge Bioscience, 1:500; 829401), α-Neurofilament-H (Biolegend, 1:100,000; PCK-592P), and α-PLP (Abcam, 1:100; ab28486), α-SERPINA3N (R&D Systems, 1:100; AF4709), α-TGFβR1 (Abcam, 1:100; ab31013). Flow cytometry: anti CD11b (PE; 1:200; 101207; BioLegend, clone M1/70), CD45 (PECy7; 1:200; 103114; BioLegend, clone 30-F11), and CD115 (APC; 1:200; 135510; BioLegend, clone AFS98)
Validation	Validation for the antibodies used for our indications are stated on the supplier websites as follows. 1) Positive signal in positive controls for α-MAG (human spleen and rat hippocampus), α-IBA (rat brain), α-CD206 (mouse lung), α-APC/CC1 (cerebellum), α-SOX9 (HepG2 cell lysate), α-GFAP (rat brain), α-TGFBR1 (human placenta). 2) α-TMEM119 was validated by absence of signal in knockout tissue. 3) References provided on supplier website for published literature using antibodies, for α-SERPINA3N, α-MBP, α-CD11b, α-CD45, α-CSF1R. 4) Validation of target by supplier via experimentation, for α-MOG (immunoblot), α-NFH (Western Blotting). For two antibodies, α-CNPase and α-PLP, the suppliers did not provide validation information. However, we have seen the same pattern of immunohistochemical signal for these myelin protein stains as for MOG, MAG, and MBP, which are all well validated. All secondary antibodies used were tested in Miron et al., 2013, Nature Neuroscience, Dillenburg et al., 2018, Acta Neuropathologica and Lloyd et al., 2019, Nature Neuroscience.

Animals and other research organisms

Policy information about studies involving animals; ARRIVE guidelines recommended for reporting animal research, and Sex and Gender in Research

Laboratory animals	This study used Csf1r-FIREd/d mice, Csf1r-FIRE+/+ mice, both of mixed background (B6CBAF1/J and C57Bl6). Mice were examined at 1, 3-4, and 6 months of age. The Plp-CreERT2 mice and Tgfbr1 fl/fl mice were both on C57Bl6J background. The offspring were assessed at 28 days of age. All mice were housed at room temperature (18-23C) at 40-60% humidity.<br>ARRIVE2 guidelines have been followed for reporting and included in the manuscript, such as ethical permissions, animal strains used, methods of termination, all commercial providers for reagents, sex of animals, exact n numbers used, statistical information and p values.
Wild animals	No wild animals were used in this study.
Reporting on sex	Both sexes were used for analysis for all animal experiments.
Field-collected samples	No field collected samples were used in this study.
Ethics oversight	Experiments were performed under a UK Home Office project licence, approved by the UK Home Office and issued under the Animals (Scientific Procedures) Act.

Note that full information on the approval of the study protocol must also be provided in the manuscript.

Flow Cytometry

Plots

Confirm that:

☒ The axis labels state the marker and fluorochrome used (e.g. CD4-FITC).

☒ The axis scales are clearly visible. Include numbers along axes only for bottom left plot of group (a 'group' is an analysis of identical markers).

☒ All plots are contour plots with outliers or pseudocolor plots.

☒ A numerical value for number of cells or percentage (with statistics) is provided.

Methodology

Sample preparation	After transcardially perfusing 10–11-week-old female mice with ice-cold PBS, brains were dissected and minced with a 22A scalpel in HBSS (without Ca2+ and Mg2+; 14175-053; Gibco) with 25 mM HEPES (10041703; Fisher Scientific). Brains were then homogenised using a Dounce homogeniser (D9938; Kimble) in HBSS (w/o Ca2+ and Mg2+) with 25 mM HEPES. Brain homogenates were separated using a 35% Percoll gradient, with centrifugation at 800 g for 20 mins at 4°C (with no brake). Cell pellets were collected and washed in PBS (w/o Ca2+ and Mg2+; 14190-094; Gibco) with 0.1% low endotoxin BSA (A8806; Sigma Aldrich). Fc receptors were blocked (1:100; 101302; BioLegend) for 15 min at 4°C on a shaker. Cells were then stained with primary antibodies.
Instrument	Data were acquired using a BD LSRFortessa™ Flow Cytometer.
Software	FCS express 7 was used for post-acquisition data analysis.
Cell population abundance	All post sort cell abundance are indicated in Extended Data Figure 1d,e.
Gating strategy	Gating strategy is provided in Extended Data Figure 1d

☒ Tick this box to confirm that a figure exemplifying the gating strategy is provided in the Supplementary Information.
